# What the COVID-19 pandemic entails for the management of patients with behavioral and psychological symptoms of dementia: experience in France

**DOI:** 10.1017/S1041610220003567

**Published:** 2020-09-15

**Authors:** Olivier Drunat, Jean Roche, Samuel Kohler, Vernaudon Julien, Saidlitz Pascal, Hermine Lenoir, Maria Soto-Martin, Alexis Lepetit, Lisette Volpe-Gillot, Vania Leclercq, Mouna Romdhani, Pierre Koskas, Florence Lebert

**Affiliations:** 1 Psycho-geriatric unit, Bretonneau Hospital, Assistance Publique Hôpitaux de Paris, Paris Nord University, France; 2 Unité Cognitivo-Comportementale, University Hospital of Lille, France; 3 Unité Cognitivo-Comportementale, Centre Mémoire Ressource et Recherche, Hospices Civils de Lyon, France; 4 Unité Cognitivo-Comportementale, University Hospital of Toulouse, France; 5 Broca Hospital, Assistance publique Hôpitaux de Paris, EA4468 Paris Descartes University, France; 6 Department of Geriatric Medicine, Gerontopole, Alzheimer Disease Research Center, Inserm UMR 1027, University Hospital of Toulouse, France; 7 Equipe mobile maladie d’Alzheimer, Institut du Vieillissement, Hospital of Charpennes, Hospices civils de Lyon, Villeurbanne, France; 8 Psycho-geriatric Unit, Léopold Bellan Hôpital, Paris, France; 9 Unité Cognitivo-Comportementale, Hôpital Rothschild, Assistance publique Hôpitaux de Paris, Sorbonne Université Paris, France; 10 Unité Cognitivo-Comportementale of Bailleul, Centre Mémoire Ressource et Recherche, University Lille Nord de France, France

The behavioral and psychological symptoms of dementia (BPSD) are well known in patients with major cognitive impairment (Bessey and Walaszek, 2019). The COVID-19 pandemic calls into question the organization of Cognitive and Behavioral Units (CBUs) specialized in the management of BPSD (Bellelli and Trabucchi, 2011; Koskas *et al.*, 2011) to limit the risk of contamination for hospitalized patients and staff. Moderate-to-severe cognitive disorders and BPSDs such as wandering and agitation explain patients’ lack of understanding of the basic protective measures against COVID-19 and their difficulty in implementing them (BMJ Best Practice, 2020). It is also difficult to find the right balance between protective but coercive measures and respect for patients’ dignity. Another difficulty is how to streamline hospital procedures to reduce workload and limit the risk of mental and physical exhaustion.

Recently, several expert committees have published guidelines on the treatment and management of patients with COVID-19 in different clinical situations (BMJ Best Practice, 2020; Wang *et al.*, 2020) but to our knowledge, none of them had focused on elderly patients with BPSD. Confronted with the urgent medical need of guidelines, a French geriatric and psychiatric task force developed recommendations based on their clinical experience in CBU. In France, CBU exists in hospitals for the purpose of managing very severe BPSD in older adults living with dementia which are systematically assessed at admission and hospital discharge by using the Neuropsychiatric Inventory. Pathological wandering is a BPSD and consists in being in constant motion, with no precise or reasonable goal. It can lead a patient with advanced dementia to involuntarily leave the hospital without medical authorization with the risk of being harmed. This specific BPSD is systematically screened prior to admission because it is usually associated with aberrant motor behavior which implies incapacity to respect barrier measures such as social distancing, wearing a mask, or sanitizing one’s hands. It is, therefore, associated with a high risk of cluster outbreak.

Depending on the stage of contamination in the unit, practical proposals are made to better manage patients with BPSD and reduce the risk of contamination for patients and hospital staff during the COVID-19 pandemic.

The stages of COVID-19 contamination in a specialized unit and the corresponding recommendations are shown in Figure 1.

**Figure 1.**
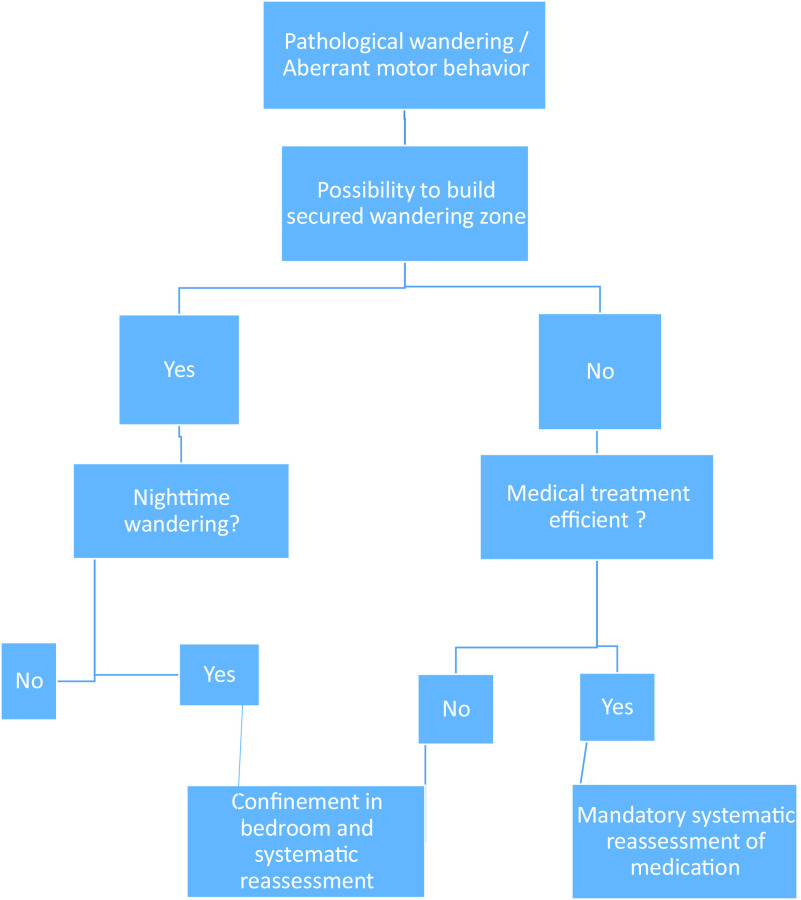
Decision tree to allow the least restrictive environment for patients who wander.

## Stage 0: No patients with COVID-19 infections in the cognitive–behavioral unit

The patients’ COVID-19 status need to be known prior to their admission in the CBU using protein chain reaction detection methods, or computed tomography scans to avoid contamination of COVID-19-free patients, bearing in mind the limits of sensitivity of each approach (BMJ Best Practice, 2020; LaHue *et al.*, 2020). No COVID-19-positive patients should be admitted to the unit as long as they are considered to be contagious (LaHue *et al.*, 2020) (minimum 24 days [Zhou, 2020] after the onset of symptoms) due to the inability of patients with BPSD to comply with protective measures. A newly admitted patient considered as COVID-19-negative should be closely screened for atypical signs evocative of COVID-19 infection such as falls, delirium, generalized weakness, dizziness, headache, rhinorrhea, conjunctivitis, chest pain, digestive symptoms, or anosmia. Isolated tachypnea or unexplained tachycardia may be warning signs too (D’Adamo *et al.*, 2020).

However, there is no argument to systematically confine wandering patients in their rooms (de Santé, 2020). Group activities should be suspended while prioritizing individual care. Staff will be cautious about the risk of transmitting COVID-19 between patients by cleaning any items used with a patient and avoiding the use of objects that are difficult to clean.

## Stage 1: Diagnostic or suspicion of a COVID-19 infection in the unit

As a first step, all COVID-19-free patients should be quickly released from the unit if possible.

Recently diagnosed COVID-19 patients will have to be transferred to a dedicated acute COVID-19 unit preferably in the same hospital while COVID-19-negative patients in the CBU should be closely monitored for signs of infection (BMJ Best Practice, 2020). These are emergency measures that take into account the rapid spread of the virus (Vanhems, 2020). Moreover, recent studies have highlighted atypical clinical presentations of COVID-19 infections in elderly patients such as delirium, psychomotor retardation, and repeated falls (Shahid *et al.,* 2020; Lin and Han, 2020).

## Stage 2: If transferring a COVID-19 patient in an acute COVID-19 unit is impossible

When there are two confirmed cases (BMJ Best Practice, 2020) or more in a unit, this unit necessarily becomes a COVID-19 unit. Therefore, only COVID-19-positive patients can be hospitalized and any discharged patient will have to be isolated by droplet isolation for at least 15 days (BMJ Best Practice, 2020). Personal protective equipment for the prevention of highly contagious diseases needs to be provided for hospital staff (Verbeek *et al.*, 2020).

The first step is to group patients together in contiguous rooms on the unit and to isolate them as much as possible in their rooms. For wandering COVID-19-positive patients, a special secure and separate section may be created within the unit to respect as far as possible the principle of freedom of movement. Indeed, grouping COVID-19-positive patients together in a protected environment can limit spreading the COVID-19 outbreak while reducing the risks related to confinement such as anxiety and depression. Single room isolation should be a second-line option and can be considered when the implementation of such a protected section is impossible for architectural, material, or workforce limitations. In this case, do not leave healthcare waste bins outside the rooms.

The second step is to reinforce the medical and paramedical staff as recommended by local health organizations. For example, the ratio of professional caregivers per patient in France is one auxiliary nurse for six patients with acute COVID-19 infections (Robert *et al.*, 2018). Moreover, staff needs to be specifically assigned to care units, either COVID-19-positive or COVID-19-negative units, in order to reduce the spread of the virus.

## Stage 3: Managing COVID-19 patients with BPSD and the hospital staff

The COVID-19 pandemic strains both staff and patients. In France, nursing assistants in CBU are qualified to manage BPSD (“Assistant de Soins en Gérontologie”) in order to limit psychotropic drug prescriptions. Nursing workforces are strengthened in COVID-19 units to enable closer monitoring of patients’ vitals and personalized care. Yet, the healthcare teams confronted with COVID-19 are particularly strained and require active psychological support from psychologists and unit managers (Bao *et al.*, 2020; Xiang *et al.*, 2020). The setting up of discussion groups and the provision of a psychological support telephone platform dedicated to healthcare professionals should be proposed (Wu *et al.*, 2009).

On the other hand, quarantine can be poorly tolerated by elderly patients, especially those with BPSD, triggering anxiety, depression, and dehydration and increase BPSD (Vanhems, 2020; Xiang *et al.*, 2020). To our knowledge, there are no therapeutic guidelines for the use of psychotropic drugs in this context. Therefore, we needed a daily multidisciplinary assessment of the benefit/risk ratio to adapt to non-pharmacological and pharmacological treatments. Non-pharmacological approaches aim to limit the isolation perceived by patients (Brooks *et al.*, 2020) and staff needs to be particularly aware of depression symptoms which are often atypical is this population (Bessey and Walaszek, 2019). This aspect of patient care should not be neglected despite the epidemic situation. Thus, staff may schedule times during the day to visit patients, help them walk around the unit or garden, offer individually assisted physical activities, or music programs in their rooms. In addition, meals are an opportunity for staff to spend time with their patients.

Since families are not allowed to visit their relatives, effective communication devices such as regular telephone or video calls could be implemented to reduce psychological stress and feelings of loneliness (Bao *et al.*, 2020; Brooks *et al.*, 2020). Furthermore, some patients may feel relief having a “transitional object” with them such as personal photos, reminders of pleasant memories. Allowing patients to wear their usual clothing also helps to preserve their sense of identity and dignity. However, due to overworked teams, families may be called upon to provide laundry services. In that case, any dirty laundry leaving the unit should be returned to the family caregiver in a water-soluble bag that is hermetically sealed (BMJ Best Practice, 2020).

Pharmaceutical treatment should be based primarily on the patient’s clinical signs (delirium, anxiety, depression, and delusion). Although benzodiazepines are often used as first-line treatments in case of acute agitation, after ruling out any somatic cause (pain, fever, urinary retention, constipation…), randomized control trials don’t support their use (McDermott and Gruenewald, 2019). On the other hand, in case of chronic agitation or anxiety, serotonin reuptake inhibitors may be prescribed, taking into account side effects such as QTc interval widening with escitalopram and citalopram (Drye *et al.*, 2012). An alternative medication for chronic agitation is Pregabalin (Supasitthumrong *et al.*, 2019). If delirium or delusion occurs, standard therapeutic algorithms can be used (McDermott and Gruenewald, 2019).

Unfortunately, in case of a drug shortage, alternative treatments must be considered. Midazolam can be used either orally or by subcutaneous injection to treat acute behavioral symptoms (McDermott and Gruenewald, 2019). When oral administration is not possible, injectable benzodiazepines are the best option, bearing in mind that diazepam, clorazepate, and clonazepam all have a long elimination half-life and can accumulate in the body if used regularly (Wu *et al.*, 2009). Injectable form of lorazepam is available in some countries and should be preferred for its shorter elimination half-life.

Good clinical practice for the prescription of psychotropic drugs (Livingston *et al.*, 2017) recommends daily assessment of the risk/benefit ratio and reducing the dosage to limit iatrogenic side effects. Fever increases the risk of side effects due to psychotropic drugs particularly antipsychotics, such as falls and dysphagia (LaHue *et al.*, 2020). Moreover, COVID-19 infections are associated with thrombogenic and arrhythmogenic risks (Guo, 2020). As with any infected frail patient, medical staff should implement nutritional supplementation (high-calorie and high-protein foods) and hydration monitoring (Lin and Han, 2020). Comprehensive geriatric assessment remains a key component to avoid usual complications such as constipation, and pain.

If chemical restraint is not possible, physical restraint may exceptionally be prescribed preferably with abdominal and pelvic straps (Livingston *et al.*, 2017). The prescription needs to specify the kind of equipment needed and the duration of validity. Patients with nighttime pathological wandering should only be confined in their rooms overnight. The medical prescription and tolerance of such restraint should be reassessed hourly. Prescriptions can, however, be anticipated through a staggered procedure. Physical restraint should be discontinued as soon as possible once the chemical restraint is effective. As always, it is important to document reasons, consent, and review procedures if physical restraint is prescribed. In some countries, physical restraint is forbidden by law, and in others, it is mandatory to obtain consent from the families or main support person.

The COVID-19 outbreak is overwhelming all healthcare systems and we need to provide new guidelines for better medical management of elderly BPSD patients and their families and preserve hospital staff by streamlining their procedures.
